# Generation of Bessel beams with tunable topological charge and polarization

**DOI:** 10.1515/nanoph-2025-0165

**Published:** 2025-06-27

**Authors:** Tong Nan, Zhiyan Zhu, Guocui Wang, Yunfei Wang, Shulin Sun, Hao Tian, Yan Zhang

**Affiliations:** School of Physics, Harbin Institute of Technology, Harbin, 150001, People’s Republic of China; Beijing Key Laboratory of Metamaterials and Devices, Key Laboratory of Terahertz Optoelectronics, Ministry of Education, Beijing Advanced Innovation Center for Imaging Theory and Technology, Department of Physics, 12379 Capital Normal University, Beijing, 100048, People’s Republic of China; Shanghai Engineering Research Centre of Ultra Precision Optical Manufacturing, Department of Optical Science and Engineering, School of Information Science and Technology, Fudan University, Shanghai, 200433, People’s Republic of China; Shanghai Key Laboratory of Metasurfaces for Light Manipulation, Fudan University, Shanghai, 200433, People’s Republic of China; Laser Micro/Nano-Fabrication Laboratory, School of Mechanical Engineering, Beijing Institute of Technology, Beijing, 100081, People’s Republic of China

**Keywords:** Bessel beams, moiré meta-device, topological charge, polarization, dynamic manipulation

## Abstract

Bessel beams hold significant potential in optical communications, particle manipulation, and medicine due to their self-healing and nondiffracting properties. However, most existing Bessel beam generation devices are either static or capable of dynamically adjusting only a single characteristic. In this paper, we propose a tunable Bessel beam generation scheme based on a moiré meta-device. The device consists of two cascaded layers of all-dielectric metasurfaces. By adjusting the relative rotation between two layers, Bessel beams with varying topological charges can be generated. Moreover, the overall rotation of the cascaded metasurfaces modulates the polarization state of the Bessel beam by leveraging both the propagation phase and geometric phase. Experimental results confirmed the generation of Bessel beams with tunable uniform linear polarization and topological charge, as well as Bessel beams with tunable topological charge and controllable polarization variations along the propagation direction. This method offers a flexible design strategy for the continuous dynamic manipulation of both the transverse and longitudinal optical field properties of Bessel beams. In addition, it may also advance the development of related fields, including optical communications, particle manipulation, and super-resolution imaging.

## 1 Introduction

Bessel beams, with their remarkable nondiffracting property, can maintain focus and intensity over extended distances, making them advantageous for applications requiring precise long-distance control and transmission. Furthermore, high-order Bessel beams can substantially enhance the information capacity and channels of optical communication systems by carrying orbital angular momentum (OAM) for information transmission [1], [2], [3], [4]. The conventional approach to generate Bessel beams involves the use of annular apertures, axicons, or spatial light modulators [1], [5], [6], [7]. However, these approaches are often limited in practical applications due to their low efficiency, bulky setups, and insufficient design flexibility. Metasurfaces, as a novel class of ultra-thin optical devices, offer a groundbreaking approach for realizing flexible, compact, and easily integrable optical modulation components [8], [9], [10], [11], [12], [13], [14], [15], [16], [17], [18], [19]. Since their emergence, numerous studies have reported the use of metasurfaces to generate intriguing Bessel beams, including the manipulation of their orbital angular momentum [20], [21], [22], polarization [23], [24], [25], [26], and propagation trajectories [27], [28], [29], [30]. Among these, switchable terahertz (THz) multi-OAM Bessel beams were developed based on a spin-decoupled reflective multifunctional metasurface, demonstrating that a single metasurface can generate multiple vortex beams with different OAMs in order to increase the information capacity of optical communication [20]. The generation of a Bessel beam with longitudinally varied polarization was demonstrated using a dielectric metasurface by leveraging circular birefringence to introduce spin-dependent wave vector differences [23]. Beyond independent OAM and polarization control of Bessel beams, a novel metasurface design was introduced to create complex optical vortices featuring on-demand tuning of both vortex strength and polarization state along the propagation path [22]. These metasurfaces enable the manipulation of different properties, promoting the versatility of Bessel beams.

Nevertheless, most of the Bessel beam generation devices mentioned above lack tunability, meaning that each device can only produce a specific type of Bessel beam. Recently, an emerging moiré metasurface, composed of two mutually rotating cascaded metasurfaces, has provided an alternative method for dynamically controlling the wavefront of electromagnetic waves. This approach has enabled the development of many interesting tunable optical devices [31], [32], [33], [34], [35], [36], [37], [38], [39], including adjustable deflectors [31], [32], variable-focus metalenses [33], [34], [35], and tunable structured beam generators [36], [37], among others. Wang et al. utilized this method to modulate the THz wavefront, achieving active control over the order and nondiffractive range of Bessel beams [36], but without involving the polarization channel. Polarization, as a crucial property of light, plays a significant role in applications such as optical communication, optical sensing, and quantum entanglement. In recent years, there has been growing interest in propagation beams with longitudinally varying polarization characteristics [40], [41], [42], [43]. Unlike the traditional beams with invariant polarization characteristics along propagation, the longitudinal varying polarization characteristics of generated beams provide new design freedom for wavefront manipulation and may expand the dimensions of many related applications. A metasurface polarization optics capable of performing parallel light processing across multiple planes was developed, enabling polarization control of Bessel beams on different planes [40]. However, the topological charge remained limited to 1st order without expansion to higher orders due to inherent design constraints. The spin-decoupled spatial partitioning method enabled the generation of longitudinally varying high-order cylindrical vector fields, achieving orders ranging from 2nd to 10th along the propagation direction [43]. Similar to the problem mentioned above, most of the generated Bessel-like beams with longitudinal polarization variations lack adjustability, that is, the polarization state or OAM corresponding to a fixed propagation distance is fixed. Moreover, the combination of longitudinally polarization transformed beams with tunable high-order Bessel beams has rarely been explored.

**Figure 1:**
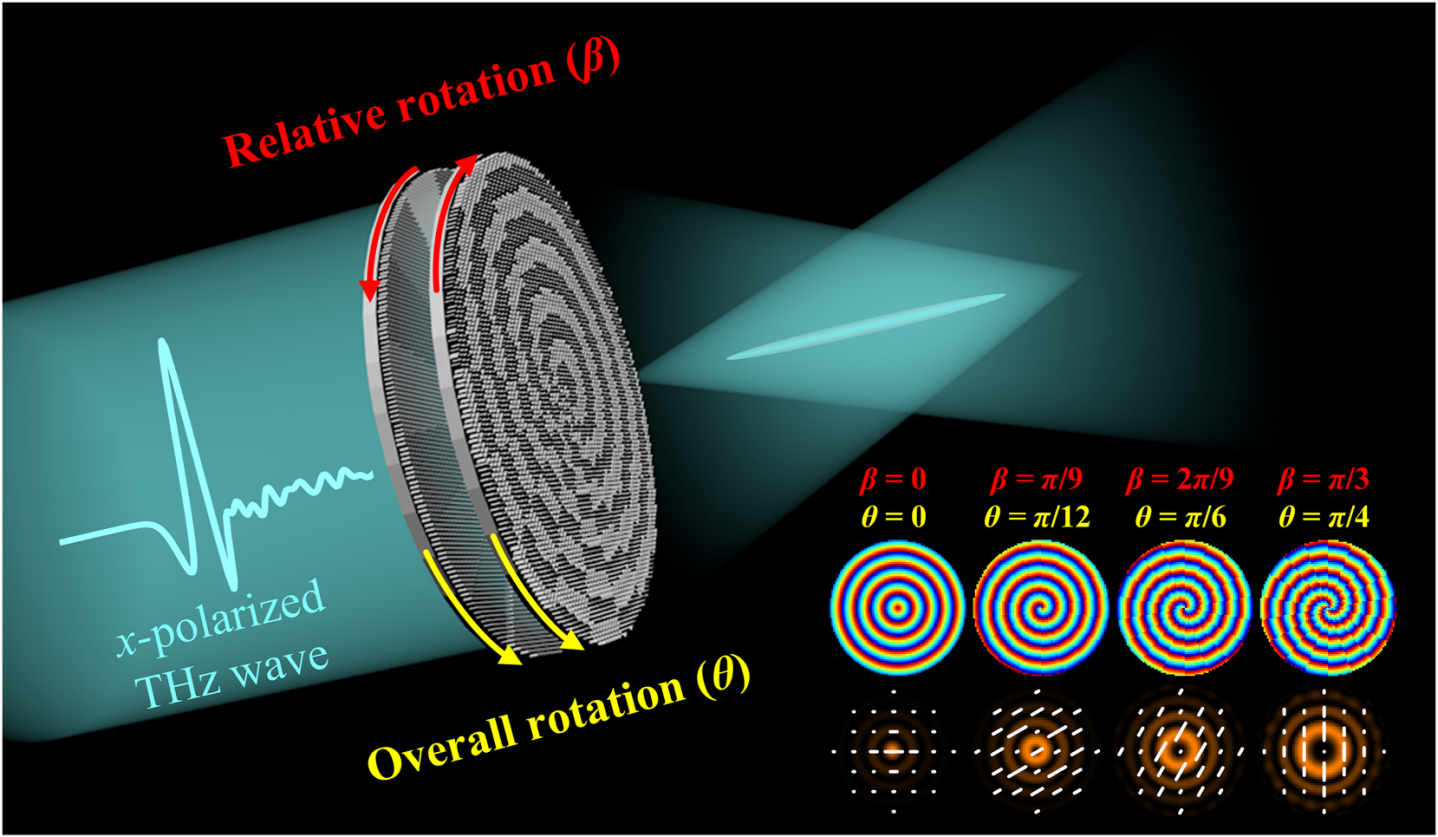
Schematic diagram of Bessel beam moiré meta-device with tunable topological charge and polarization. The incident beam is *x*-polarized.

In this paper, we combine geometric and propagation phases to simultaneously modulate phase and polarization, constructing a THz-band moiré meta-device with cascaded all-dielectric metasurfaces that can dynamically generate Bessel beams with tunable topological charge and polarization, as illustrated in Figure 1. To validate the feasibility of our proposed method, we fabricated samples with a uniform polarization distribution and by adjusting the relative and overall rotation angles of the two metasurface layers, experimentally generated Bessel beams with tunable topological charge and linear polarization states. Furthermore, since the polarization state of the Bessel beam in the direction of propagation is related to the distribution of radially polarized states in the plane of generation, we fabricated a second set of samples with a nonuniform polarization distribution. When a linearly polarized beam passes through this meta-device, the polarization state of the output beam undergoes longitudinal rotation in free space, enabling the experimental generation of Bessel beams with tunable topological charge and controllable polarization variations along the propagation direction.

## 2 Principle and design method

The working principle of the moiré metasurface based on relative rotation is as follows: By cascading two layers of metasurfaces with carefully designed phase responses, the relative rotation between them induces a moiré pattern, which dynamically modulates the wavefront of transmitted light. To achieve dynamic control of Bessel beams using a moiré metasurface, we assume that the phase distributions of two cascaded metasurfaces in the polar coordinate system are defined as $\varphi_{1}\left(r,\alpha \right)=\alpha^{2},\varphi_{2}\left(r,\alpha \right)=-\alpha^{2}+2\pi /\lambda r\sin\gamma$ , where *r* is the radial coordinate and *α* is the angular coordinate ranging from 0 to 2π. When one metasurface is rotated by an angle *β* relative to the other, the total phase distribution can be expressed as follows [20]:

(1) $$\varphi_{\text{joint}}=\varphi_{1}\left(r,\alpha \right)+\varphi_{2}\left(r,\alpha -\beta \right)=2\beta \alpha -\beta^{2}+\frac{2\pi }{\lambda }r\sin\gamma$$

High-order Bessel beams can be generated using a combination of a vortex phase plate and an axicon, with the corresponding total phase distribution given by:

(2) $$\varphi_{\text{total}}=l\alpha +\frac{2\pi }{\lambda }r\sin\gamma$$

*l* is the order of the high-order Bessel beam, *γ* is the base angle of the axicon and determines the length of the nondiffraction region. Compared with Equation (1), it can be found that the order of the high-order Bessel beam, *l* = 2*β*, and *β* ^2^ is the overall phase delay term uniformly distributed in space. Thus, by adjusting the relative rotation angle *β* between the two layers of the metasurface in the moiré structure, the order of high-order Bessel beams can be easily controlled. However, the 2π ambiguity of rotation inevitably induces a sectoring effect in the moiré meta-device, which reduces its efficiency and compromises the quality of the focused beam. This issue can be resolved through phase quantization and compensation [44]. The angle range [0, 2π) is partitioned into *N* equal sectors (where *N* is an even integer) with an angular step of Δ*α*, such that *N* = 2π/Δ*α*. Subsequently, the phase profiles of both metasurfaces are discretized using this step Δ*α*, where phase ambiguities within each sector can be resolved by applying phase corrections in integer multiples of 2π. Following this discretization process, the phase distributions of the two metasurfaces can be mathematically represented as

(3) $$\varphi_{1}\left(r,m\right)=m^{2}\frac{\pi }{N}$$

(4) $$\varphi_{2}\left(r,m\right)=-m^{2}\frac{\pi }{N}+\frac{2\pi }{\lambda }r\sin\gamma$$

(5) $$\begin{array}{cc} \varphi_{\text{joint}} & =\frac{\pi }{N}{\left(\frac{\alpha }{\Delta \alpha }\right)}^{2}-\frac{\pi }{N}{\left(\frac{\alpha -\beta }{\Delta \alpha }\right)}^{2}+\frac{2\pi }{\lambda }r\sin\gamma \\ & =n\alpha -n^{2}\frac{\pi }{N}+\frac{2\pi }{\lambda }r\sin\gamma \end{array}$$

where *m* is an integer, expressed as *m* = *α*/Δ*α*. The value of *N* in this paper is selected as 18. The relative rotation angle *β* between the two metasurface layers must be an integer multiple of Δ*α*, expressed as: *β* = *n*Δ*α*, where *n* is an integer satisfying -*N*/2 ≤ *n* < *N*/2. Comparing Equation (5) with the phase distribution of a high-order Bessel beam, the topological charge *l* can be modulated by the number of *N* and the mutual rotation *β*, giving the following expression

(6) $$l=n=N\frac{\beta }{2\pi }$$

The above derivation and implementation demonstrate that the topological charge of the Bessel beam can be dynamically controlled by cascading two metasurfaces with specially designed phase distributions and rotating them relative to each other. To achieve simultaneous dynamic control of both topological charge and polarization state, it is essential to design metasurface units with spin-decoupling functionality. This requires incorporating both propagation phase and geometric phase into the metasurface design. The phase change resulting from the interaction between the metasurface structure and the incident light can be described as the superposition of the propagation phase and the geometric phase [45], [46]:

(7) $$\phi_{LR}=\varphi_{d}-2\theta$$

(8) $$\phi_{RL}=\varphi_{d}+2\theta$$

*ϕ*_*LR*_ (*ϕ*_*RL*_) represents the phase change of the transmitted light relative to the incident light when the incident light is right-handed circularly polarized (left-handed circularly polarized) and the transmitted light is left-handed circularly polarized (right-handed circularly polarized). Here, *φ*_*d*_ denotes the propagation phase, and *θ* is the rotation angle of the metasurface unit. A linearly polarized incident light with amplitude *E* and polarization orientation angle *θ*_0_ (the angle between the polarization direction and the *x*-axis) can be decomposed into left-handed and right-handed circularly polarized components of equal amplitude:

(9) $$E_{\text{in}}=\frac{E}{2}{\text{e}}^{\text{i}\theta_{0}}\left[\begin{array}{c} 1\\ -i \end{array}\right]+\frac{E}{2}{\text{e}}^{-\text{i}\theta_{0}}\left[\begin{array}{c} 1\\ i \end{array}\right]$$

The transmitted left-handed and right-handed circularly polarized light components are transformed as follows:

(10) $$E_{LR}=A\frac{E}{2}{\text{e}}^{\text{i}\theta_{0}}{\text{e}}^{\text{i}\phi_{LR}}\left[\begin{array}{c} 1\\ i \end{array}\right]$$

(11) $$E_{RL}=A\frac{E}{2}{\text{e}}^{-\text{i}\theta_{0}}{\text{e}}^{\text{i}\phi_{LR}}\left[\begin{array}{c} 1\\ -i \end{array}\right]$$

The total transmitted field is given by:

(12) $$E_{t}=E_{LR}+E_{RL}=AE{\text{e}}^{\text{i}\phi_{d}}\left[\begin{array}{c} \cos\left(2\theta -\theta_{0}\right)\\ \sin\left(2\theta -\theta_{0}\right) \end{array}\right]$$

In the ideal case where the metasurface unit acts as a perfect half-wave plate, the two circularly polarized components of the incident light will be completely converted into orthogonal circularly polarized components at the transmission end after interacting with the metasurface. These converted components then recombine at the transmission end, forming a new linearly polarized light.

**Figure 2:**
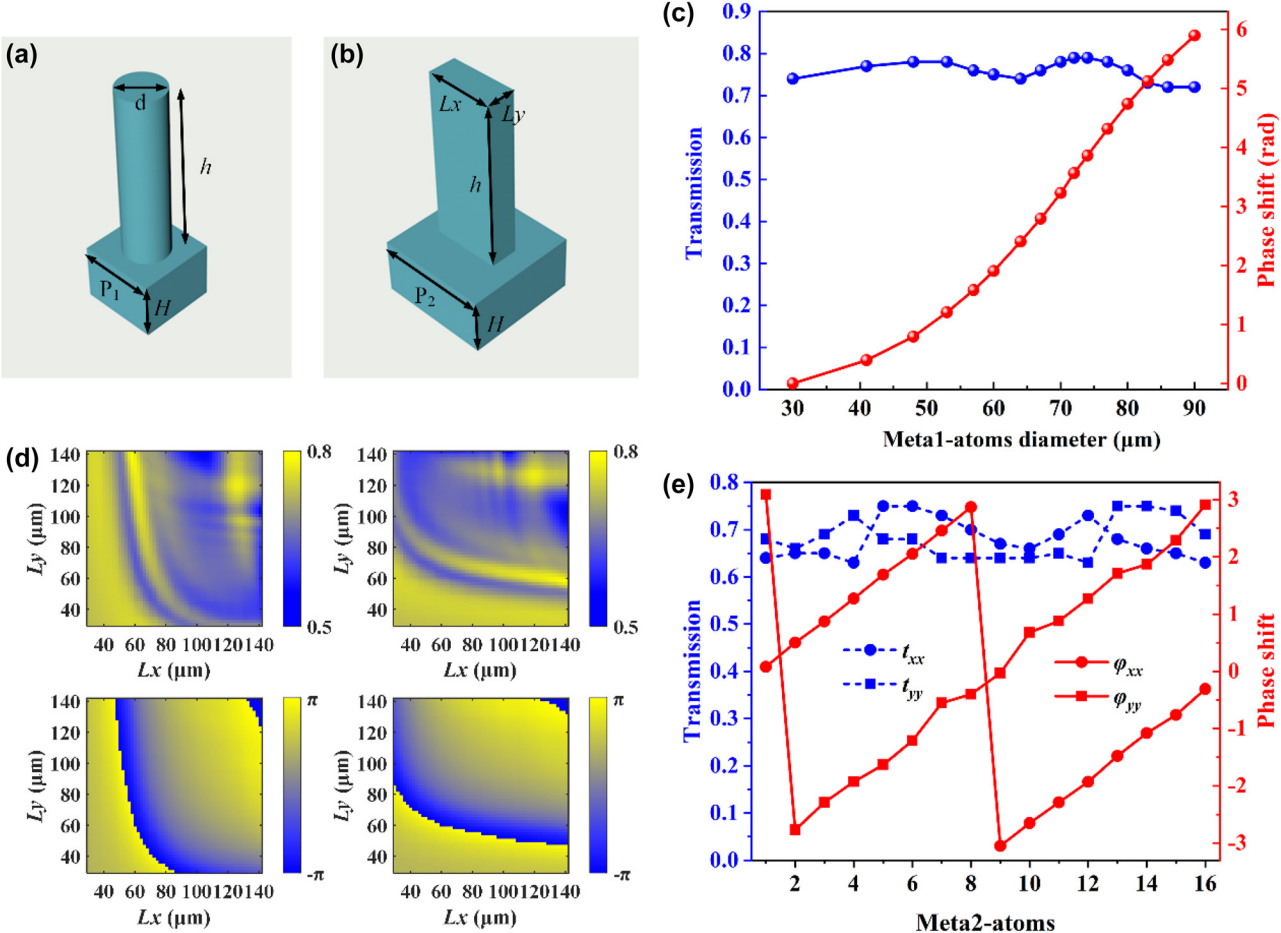
Structural schematic and simulation results. (a), (b) Schematic of the proposed silicon circular pillars and rectangular pillars unit cells. (c) Amplitudes and phase responses of the selected 16 meta1-atoms. (d) Transmission amplitudes and phase shifts of the meta2-atoms with different geometric sizes under *x*-polarized incidence and *y*-polarized incidence. (e) Amplitudes and phase responses of the selected 16 meta2-atoms.

To achieve copolarization phase modulation and spin-decoupling functionalities, we have designed two types of metasurface unit cells: meta1’s silicon circular pillars and meta2’s silicon rectangular pillars, both operating at the frequency of 0.9 THz. The propagation characteristics of the two types of silicon pillars were simulated using the finite difference time domain (FDTD) method. Periodic boundary conditions were applied in the *X* and *Y* directions, while a perfectly matched layer (PML) boundary was employed in the *Z* direction. As shown in Figure 2a, the period P_1_ of the silicon circular pillars is 100 μm, the height *h* is 200 μm, and the substrate thickness *H* is 300 μm. By changing the diameter *d* of the circular pillars, their transmission amplitude and phase shift are simulated. Here, sixteen meta1-atoms are selected, and their corresponding amplitudes and phase shifts are plotted in Figure 2c. The structural parameters for these meta1-atoms are listed in Table 1 (Appendix A). It can be seen that the transmission amplitude remains around 0.75 and their phase shift covers a range of 2π at an interval of π/8. For the silicon rectangular pillars, as shown in the Figure 2b, P*2* is 150 μm, *h* is 200 μm, and *H* is 300 μm. By changing the length *Lx* and width *Ly* of the rectangular column, the corresponding transmission amplitude and phase shift are simulated. The silicon rectangular pillars not only need to meet the conditions of the half-wave plate (for the transmitted orthogonal copolarization components, the amplitude is equal and the phase difference is π) to achieve polarization state adjustment but also need to meet the dynamic phase that can evolve in the range of 0∼2π to achieve the required phase distribution. As shown in Figure 2d, we have established a database of the transmission amplitude and phase shift (*t*_*xx*_, *t*_*yy*_, *φ*_*xx*_, *φ*_*yy*_) as the size of the rectangular column (*Lx*, *Ly*) changes when the incident light frequency is 0.9 THz. Here, *t*_*xx*_ (*t*_*yy*_) and *φ*_*xx*_ (*φ*_*yy*_) represent the transmission amplitude and phase shift of the transmitted *x*-polarization (*y*-polarization) component under the incident *x*-polarization (*y*-polarization) light, respectively. The structural parameters for these meta2-atoms are listed in Table 2 (Appendix A).

## 3 Results and discussion

**Figure 3:**
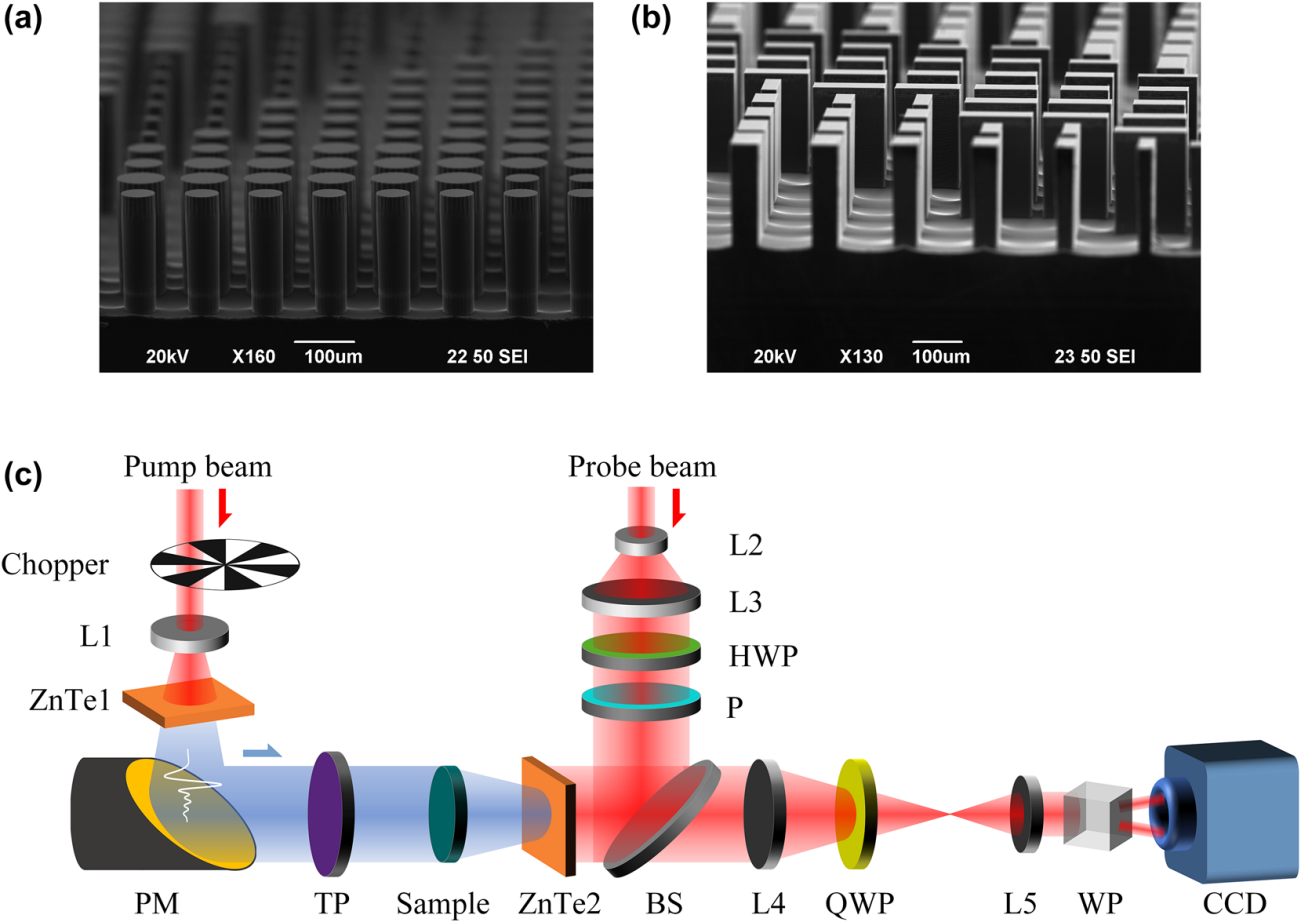
SEM images of fabricated samples and experimental setup. (a), (b) SEM images of a part of the fabricated meta1 and meta2. (c) Schematics of the THz focal-plane imaging system. (L – lens; PM – parabolic mirror; TP – THz polarizer; HWP – half wave plate; P – polarizer; BS – beam splitter; QWP – quarter-wave plate; WP, Wollaston prism; CCD – charge coupled device).

**Figure 4:**
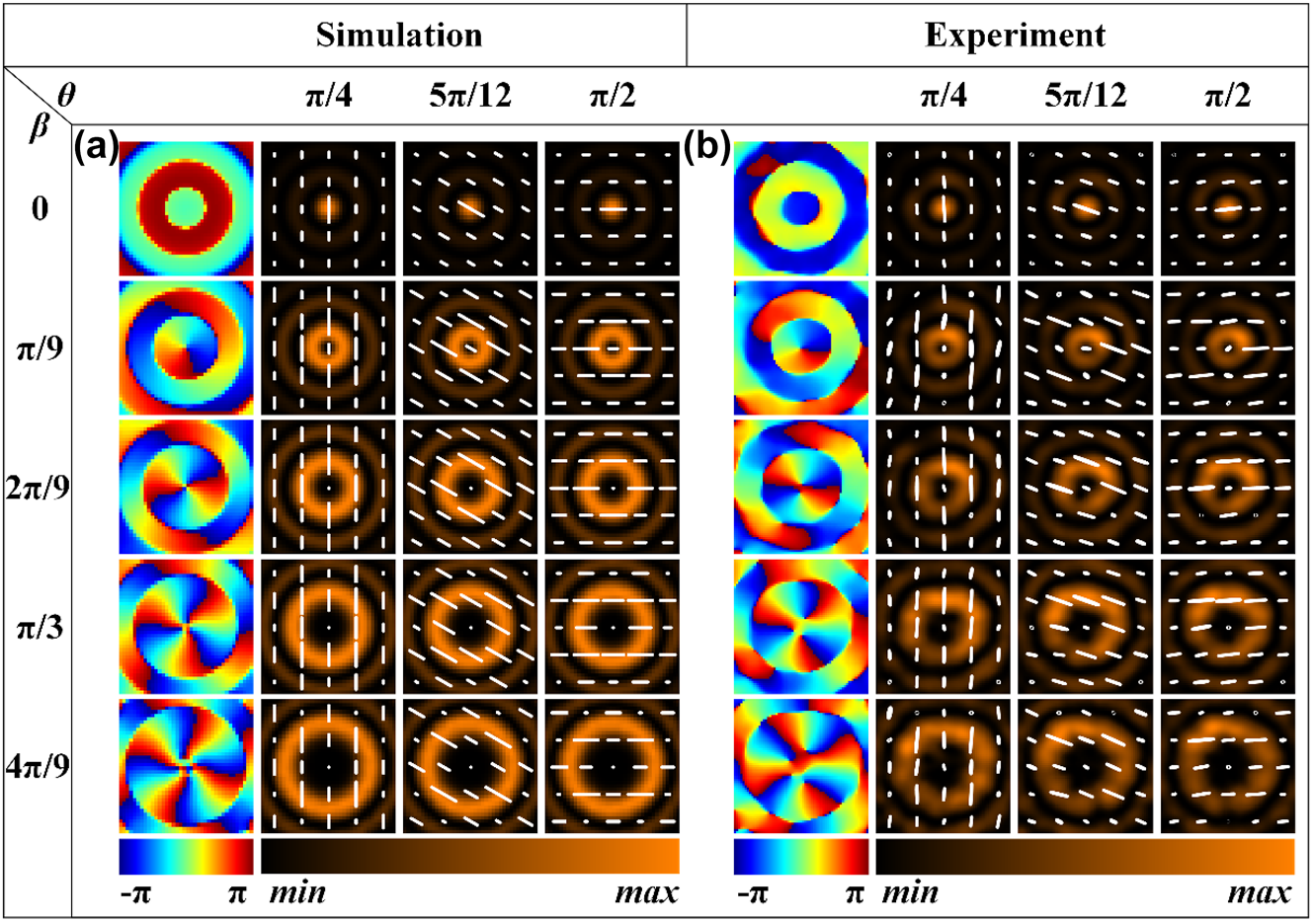
Simulation and experimental results of Bessel beams with tunable topological charge and polarization. (a), (b) Simulation and experimental results of generated Bessel beams’ field distribution in the *x–y* plane at a propagation distance *z* of 12 mm when the relative rotation angle between meta1 and meta2 is 0, π/9, 2π/9, π/3, and 4π/9, with an overall rotation of π/4, 5π/12, and π/2, respectively. The solid white line represents the polarization state.

To verify the feasibility of our proposed solution, two sets of samples were designed, fabricated, and characterized. Scanning electron microscope images of a part of the fabricated meta1 and meta2, fabricated using UV lithography and Bosch etching process technology, are shown in Figure 3a and b. It is not surprising that the circular and rectangular outlines of the blocks contained in the meta-arrays are well defined and the sidewalls are quite steep. For more detailed information on the sample preparation, see Experimental Section (Appendix B). The meta1 of both sample sets were identical. In the first group, the meta2 consisted of rectangular silicon pillars that were uniformly distributed, with the initial orientation of the rectangular pillars is set to 0°, enabling the generation of Bessel beams with arbitrary linear polarization and topological charges of integer orders ranging from 0 to 4. Figure 4a presents the simulation results of the generated Bessel beams’ field distribution in the *x*-*y* plane at a propagation distance *z* of 12 mm. It can be observed that under *x*-linearly polarized incidence, with the relative rotation angle *β* between the two metasurfaces increasing in π/9 steps from 0 to 4π/9, Bessel beams of orders 0 to 4 are generated. Furthermore, when the overall rotation angles *θ* of the two metasurfaces are π/4, 5π/12, and π/2, the polarization states of the Bessel beams correspond to 90°, 150°, and 180° linear polarization, respectively. Initially, we mounted the two metasurfaces, meta1 and meta2, onto a calibrated and rotatable cage sample holder, with both meta1 and meta2 set at an initial angle of 0°. The assembled holder was then placed on a motorized translation stage in front of the detection crystal of the THz focal-plane imaging system, as shown in Figure 3c. The detailed measuring steps can be found in the Experimental Section too. The translation stage was controlled to scan and image from 3 mm to 20 mm away from the detection crystal in 0.5 mm steps, capturing the complex amplitude distribution of the Bessel beam at various *z* positions. Rotated meta2 to adjust the relative rotation angle between meta1 and meta2 to 0, π/9, 2π/9, π/3, and 4π/9. Then, using the angle of meta2 as a reference, rotated the two metasurfaces simultaneously so that the angle corresponding to meta2 was 0. Subsequently, rotated the two metasurfaces as a whole by π/4, 5π/12, and π/2. Each relative rotation angle corresponded to three overall rotations, during which the complex amplitudes *Ex* and *Ey* were measured and synthesized. Figure 4b presents the experimental results of the field distribution on the *x*-*y* plane for meta2 at a distance of 12 mm from the detection crystal, under varying relative and overall rotation angles. It can be observed that when the relative rotation angle between meta1 and meta2 is adjusted to 0, π/9, 2π/9, π/3, and 4π/9, with an overall rotation of π/4, Bessel beams of orders 0, 1, 2, 3, and 4 are generated, respectively, all exhibiting a 90° linear polarization state. When the overall rotation is set to 5π/12 and π/2, the polarization states of the generated Bessel beams of different orders change to 150° and 180° linear polarization, respectively. The experimental results are in good agreement with the simulations and align with the theoretical derivation, which states that the linear polarization state changes by 2*θ* for an overall rotation of *θ*. However, there is a slight discrepancy between the measured polarization and the corresponding simulated polarization, which may be attributed to experimental errors introduced by rotating the sample. In addition, the simulation and experimental results of generated Bessel beams’ field distribution in the *x*-*z* plane when the relative rotation angle between meta1 and meta2 is 0, π/9, 2π/9, π/3, and 4π/9, with an overall rotation of π/2, can be found in Figure 7. (Appendix C).

**Figure 5:**
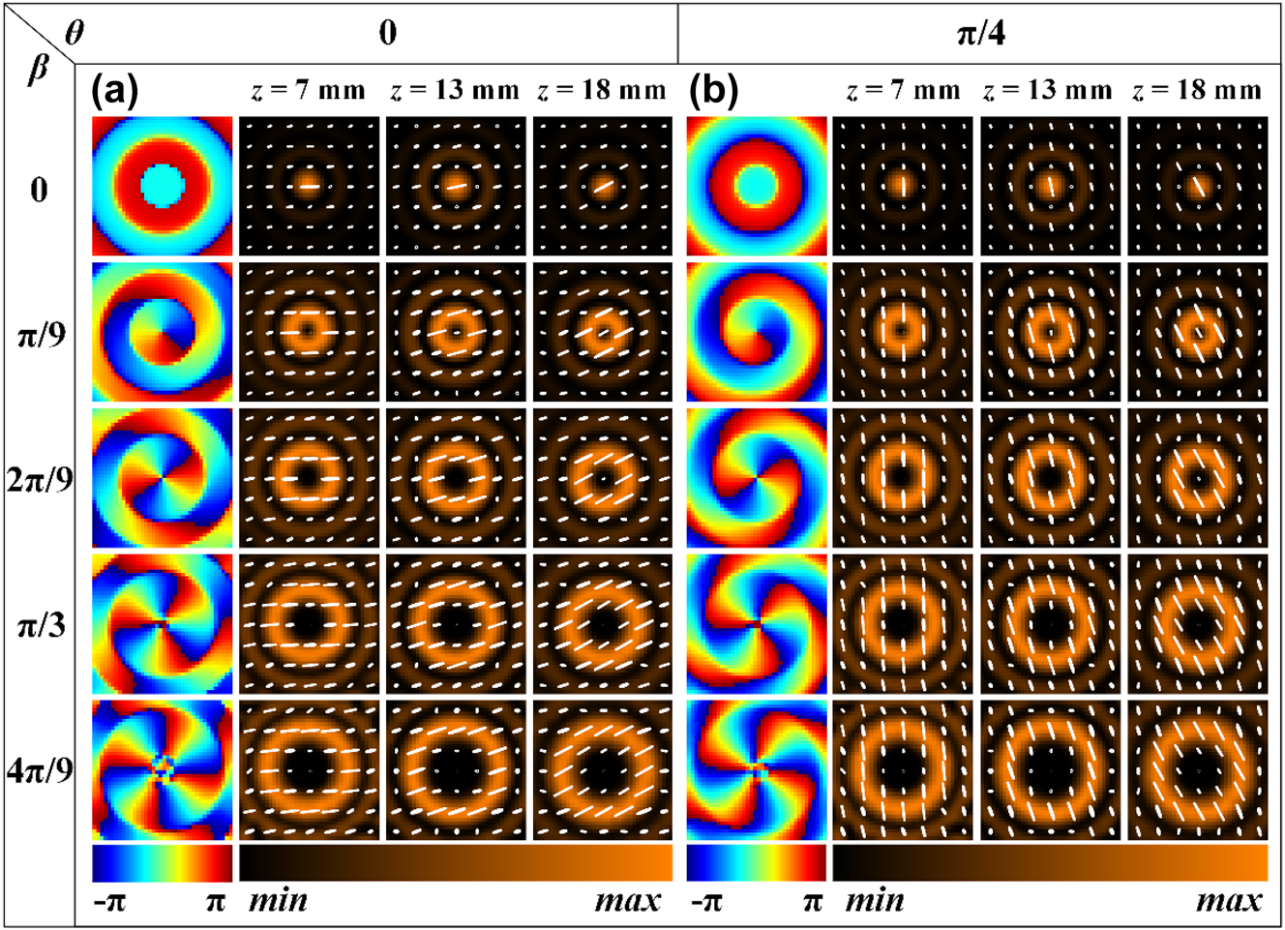
Simulation results of Bessel beams with tunable topological charge and propagation-dependent polarization. (a), (b) Simulation results of the *x-y* plane field distribution of the generated Bessel beams at different propagation distances when the relative rotation angle between meta1 and meta2 is 0, π/9, 2π/9, π/3, and 4π/9, with an overall rotation of 0 and π/4, respectively.

**Figure 6:**
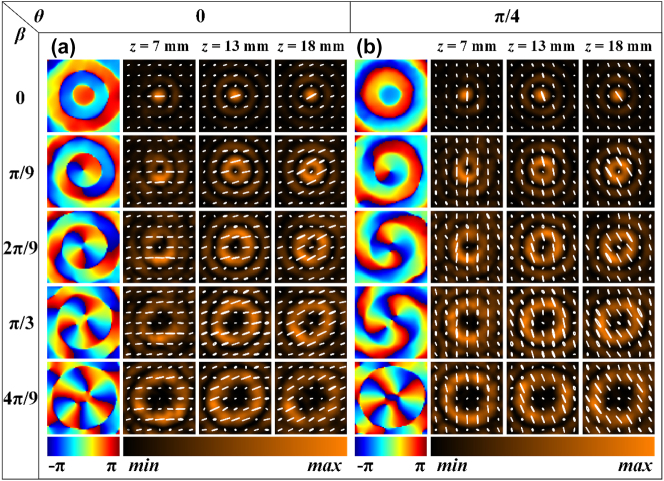
Experimental results of Bessel beams with tunable topological charge and propagation-dependent polarization. (a), (b) Experimental results of the *x-y* plane field distribution of the generated Bessel beams at different propagation distances when the relative rotation angle between meta1 and meta2 is 0, π/9, 2π/9, π/3, and 4π/9, with an overall rotation of 0 and π/4, respectively.

**Figure 7:**
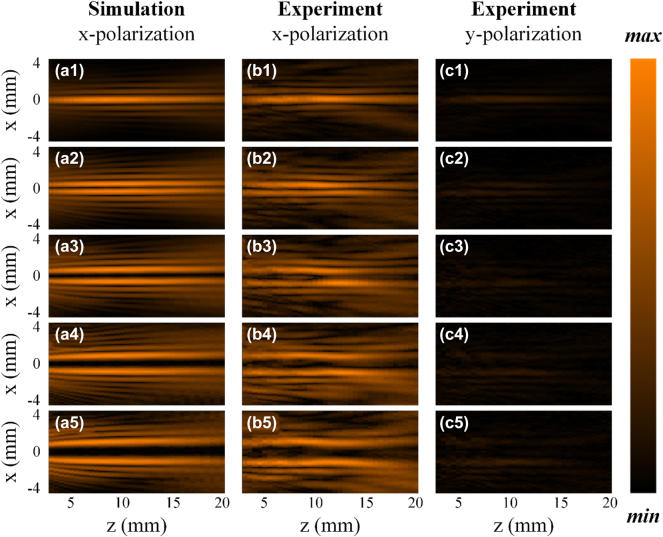
Simulation and experimental results of generated Bessel beams’ field distributions in the *x*-*z* plane when the relative rotation angle between meta1 and meta2 is 0, π/9, 2π/9, π/3, and 4π/9, with an overall rotation of π/2. (a1–a5) Simulation results of generated Bessel beams’ *Ex* field distributions. (b1–b5) and (c1–c5) Experimental results of generated Bessel beams’ *Ex* and *Ey* field distributions, respectively.

**Figure 8:**
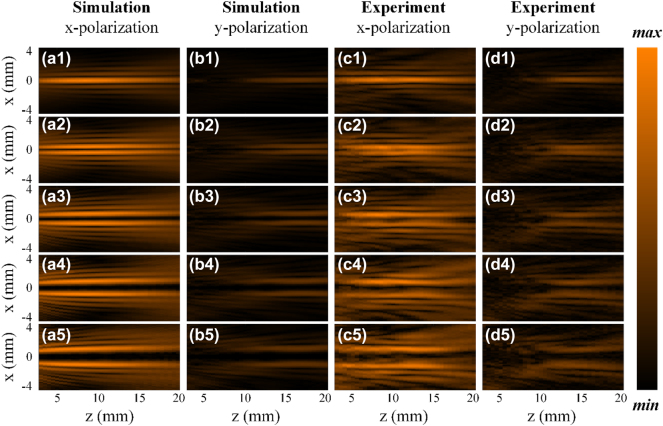
Simulation and experimental results of generated Bessel beams’ field distributions in the *x*-*z* plane when the relative rotation angle between meta1 and meta2 is 0, π/9, 2π/9, π/3, and 4π/9, with an overall rotation of 0. (a1–a5) and (b1–b5) Simulation results of generated Bessel beams’ *Ex* and *Ey* field distributions in the *x*-*z* plane, respectively. (c1–c5) and (d1–d5) Experimental results of generated Bessel beams’ *Ex* and *Ey* field distributions, respectively.

**Figure 9:**
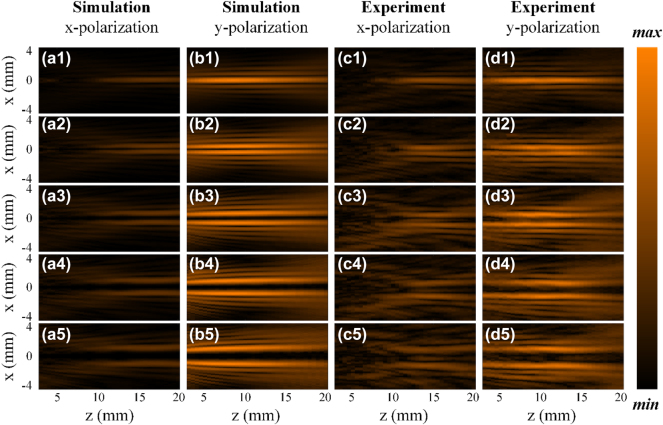
Simulation and experimental results of generated Bessel beams’ field distribution in the *x*-*z* plane when the relative rotation angle between meta1 and meta2 is 0, π/9, 2π/9, π/3, and 4π/9, with an overall rotation of π/4. (a1–a5) and (b1–b5) Simulation results of generated Bessel beams’ *Ex* and *Ey* field distribution in the *x*-*z* plane, respectively. (c1–c5) and (d1–d5) Experimental results of generated Bessel beams’ *Ex* and *Ey* field distributions in the *x*-*z*, respectively.

The rectangular silicon pillars of meta2 in the second group of samples are nonuniformly distributed, enabling the generation of Bessel beams with arbitrary integer orders from 0 to 4, where the polarization varies with the propagation distance. Specifically, within a radius range of 0–6 mm for meta2, rectangular silicon pillars with rotation angles of -π/36, 0, π/36, π/18, π/12, and π/9 are arranged at 1 mm intervals. Figure 5a presents the simulation results of the *x*-*y* plane field distribution of the generated Bessel beams at different propagation distances when the overall rotation angle is 0. The phase corresponds to a propagation distance *z* of 13 mm. It can be observed that under *x*-linear polarization incidence, with the relative rotation angle *β* between the two metasurfaces increasing in π/9 steps from 0 to 4π/9, Bessel beams of orders 0 to 4 are generated. Furthermore, the polarization varies at different propagation distances *z*: specifically, at *z* = 7 mm, 13 mm, and 18 mm, the polarization corresponds to 0°, 15°, and 30° linear polarization, respectively. Figure 5b displays the simulation results of the *x*-*y* plane field distribution of the generated Bessel beams at different propagation distances when the overall rotation angle is π/4. In this case, Bessel beams of orders 0 to 4 are generated, with the polarization at *z* = 7 mm, 13 mm, and 18 mm corresponding to 90°, 105°, and 120° linear polarization, respectively. Similarly, meta1 and meta2 were fixed onto a rotatable cage sample holder, which was then placed on a translation stage in front of the detection crystal of the THz focal plane imaging system. The translation stage was controlled to scan within a range of 3 mm–20 mm from the detection crystal, with a step size of 0.5 mm, to obtain the complex amplitude distribution of the Bessel beams at different *z* positions. The rotation method for the two metasurfaces was the same as that used for the first group. Figure 6a presents the experimental results of the *x*-*y* plane field distribution of the generated Bessel beams at different propagation distances when the overall rotation angle is 0. It can be observed that when the relative rotation angle between meta1 and meta2 is adjusted to 0, π/9, 2π/9, π/3, and 4π/9, Bessel beams of orders 0, 1, 2, 3, and 4 are generated, respectively. Additionally, the polarization state varies with the propagation distance: specifically, at *z* = 7 mm, 13 mm, and 18 mm, the polarization corresponds to 0°, 15°, and 30° linear polarization, respectively. Figure 6b presents the experimental results of the *x*-*y* plane field distribution of the generated Bessel beams at different propagation distances when the overall rotation angle is π/4. It can be observed that when the relative rotation angle between meta1 and meta2 is adjusted to 0, π/9, 2π/9, π/3, and 4π/9, Bessel beams of orders 0, 1, 2, 3, and 4 are generated, respectively. Furthermore, the polarization state varies with the propagation distance: specifically, at *z* = 7 mm, 13 mm, and 18 mm, the polarization corresponds to 90°, 105°, and 120° linear polarization, respectively. In addition, the simulation and experimental results of generated Bessel beams’ field distribution in the *x*-*z* plane when the relative rotation angle between meta1 and meta2 is 0, π/9, 2π/9, π/3, and 4π/9, with an overall rotation of 0 and π/4, can be found in Figures 8 and 9, respectively (Appendix D).

## 4 Conclusions

In summary, we have demonstrated a Bessel beams generation scheme with controllable topological charge and polarization based on all-dielectric moiré meta-devices. A two-layer cascaded metasurface was constructed using propagation phase and geometric phase. The wavefront and polarization state were independently and dynamically controlled through relative rotation and overall rotation between the two layers of the metasurface. We designed two samples in the THz band for experimental verification and generated Bessel beams with adjustable topological charge and uniform polarization, as well as Bessel beams with tunable topological charge and controllable polarization variations along the propagation direction. The experimental results are in good agreement with the simulations. We believe that this simple and effective scheme for flexibly manipulating the characteristics of Bessel beams provides an effective strategy for continuous manipulation of beams and has the potential to find applications in optical communications, particle manipulation, and super-resolution imaging.

## Appendix A: Structural parameters of selected meta-atoms

See Appendix Tables 1 and 2.

**Table 1:** Structural parameters of selected meta1-atoms.

Number	Diameter *d*/μm	Amplitude	Phase	Number	Diameter *d*/μm	Amplitude	Phase
1	30	0.74	0	9	70	0.78	3.228
2	41	0.77	0.394	10	72	0.79	3.563
3	48	0.78	0.792	11	74	0.79	3.860
4	53	0.78	1.205	12	77	0.78	4.313
5	57	0.76	1.583	13	80	0.76	4.739
6	60	0.75	1.905	14	83	0.73	5.123
7	64	0.74	2.405	15	86	0.72	5.480
8	67	0.76	2.794	16	90	0.72	5.896

**Table 2:** Structural parameters of selected meta2-atoms.

Number	Length *Lx*/μm	Width *Ly*/μm	*x*-amplitude	*x*-phase	*y*-amplitude	*y*-phase
1	104	50	0.64	0.08	0.68	3.09
2	114	52	0.65	0.50	0.66	−2.77
3	124	54	0.65	0.87	0.69	−2.29
4	138	54	0.63	1.27	0.73	−1.93
5	30	114	0.75	1.69	0.68	−1.63
6	40	94	0.75	2.05	0.68	−1.21
7	46	94	0.73	2.46	0.64	−0.55
8	50	92	0.7	2.87	0.64	−0.40
9	52	98	0.67	−3.05	0.64	−0.03
10	52	120	0.66	−2.65	0.64	0.68
11	54	124	0.69	−2.29	0.65	0.88
12	54	138	0.73	−1.93	0.63	1.27
13	118	30	0.68	−1.48	0.75	1.71
14	104	36	0.66	−1.08	0.75	1.87
15	94	44	0.65	−0.76	0.74	2.29
16	94	50	0.63	−0.31	0.69	2.91

## Appendix B: Experimental section

*Experimental Setup*: The schematic diagram of the THz focal-plane imaging system is shown in the Figure 3c. The light source used is a femto-second laser amplifier with an 800 nm central wavelength, a 35-fs pulse duration, a 1 kHz repetition rate. The laser pulse is divided into two beams by a nonpolarizing beam splitter, including pump beam and probe beam. The pump beam with an average power of 1.8 W is expanded by a concave lens (L1) and irradiated onto a zinc-telluride crystal (ZnTe1) oriented in the <110> direction, and THz radiation is excited via the optical rectification effect. A THz polarizer is used to maintain the polarization of illuminating beam. The THz pulse is collimated and irradiated to the sample through a gold-plated parabolic mirror (PM). The THz pulse carrying the sample information is incident on another zinc-telluride crystal (ZnTe2) oriented in the <110> direction. The probe beam with an average power of 0.2 W is first expanded by a concave lens (L2) and a convex lens (L3), and its central part is used for imaging measurement. Subsequently, the probe beam passes through a half wave plate (HWP) and a polarizer. The HWP is used to adjust the polarization state of the probe beam, and the polarizer is used to maintain polarization. The polarization of the probe beam reflected by ZnTe2 is modulated by the THz electric field through the Pockels effect, and the imaging module consisted of a quarter wave plate (QWP), a Wollaston prism (WP), two convex lenses (L4 and L5), and a CCD camera. In order to improve the signal-to-noise ratio of the system, a dynamic subtraction technique is used to reduce the impact of laser power fluctuations [47]. A mechanical chopper with a 25 Hz frequency was placed in the pump beam path to modulate the output of the THz pulse and synchronized with the CCD for image acquisition.

*Sample Fabrication*: All silicon-based samples are prepared using UV lithography and Bosch etching process technology. In the first step, after cleaning the sample, we bake the silicon wafer on a hot plate at 180 °C to fully remove the moisture on the surface of the silicon wafer, and then spin-coat HMDS on it. These two processes ensure that the sample pattern has a good morphology after the silicon wafer is etched. Then, we spin-coat Az4620 photoresist and use standard photolithography process to realize the transfer of the pattern from the design drawing to the photolithography pattern on the wafer. Then, we use the Bosch etching process to achieve the precise transfer of the structure from the photolithography pattern to the actual device structure. After etching, the sample is cleaned by water bath heating and plasma stripping machine to obtain a relatively perfect sample.

## Appendix C: Simulation and experimental results of the field distributions of the Bessel beams generated by the samples with uniform polarization distribution in the *x*-*z* plane

Figure 7 presents the simulation results of generated Bessel beams’ field distributions by the samples with uniform polarization distribution in the *x*-*z* plane when the relative rotation angle between meta1 and meta2 is 0, π/9, 2π/9, π/3, and 4π/9, with an overall rotation of π/2. Bessel beams of orders 0, 1, 2, 3, and 4 are generated when the relative rotation angle between meta1 and meta2 is 0, π/9, 2π/9, π/3, and 4π/9, with an overall rotation of π/2, the simulation results are shown in the Figure 7a1–a5, respectively. The generated Bessel beams are *x*-polarized when the overall rotation is π/2. Figure 7b1–b5 and c1–c5 present the corresponding experimental results of generated Bessel beams’ *Ex* and *Ey* field distributions in the *x*-*z* plane. It can be found that the electric field energy is mainly distributed in the *x*-polarization, while the *y*-polarization distribution is very weak, which is basically consistent with the expectation.

## Appendix D: Simulation and experimental results of the field distributions of the Bessel beams generated by the samples with nonuniform polarization distribution in the *x*-*z* plane.

Figure 8 presents the simulation and experimental results of generated Bessel beams’ field distribution by the samples with nonuniform polarization distribution in the *x*-*z* plane when the relative rotation angle between meta1 and meta2 is 0, π/9, 2π/9, π/3, and 4π/9, with an overall rotation of 0. Figure 8a1–a5 and b1–b5 present the simulation results of generated Bessel beams’ *Ex* and *Ey* field distributions in the *x*-*z* plane, respectively. The polarization changes with different propagation distances. Specifically, the polarization changes from 0° linear polarization to 30° linear polarization in the range of *z* = 7 mm to *z* = 18 mm. It can be observed that the *x* polarization gradually decreases while the *y* polarization gradually increases with the transmission distance. Figure 8c1–c5 and d1–d5 show the experimental results of generated Bessel beams’ *Ex* and *Ey* field distributions in the *x*-*z* plane, respectively, which is basically consistent with the simulation results. Figure 9 presents the simulation and experimental results of generated Bessel beams’ field distributions by the samples with nonuniform polarization distribution in the *x*-*z* plane when the relative rotation angle between meta1 and meta2 is 0, π/9, 2π/9, π/3, and 4π/9, with an overall rotation of π/4. Figure 9a1–a5 and b1–b5 present the simulation results of generated Bessel beams’ *Ex* and *Ey* field distributions in the *x*-*z* plane, respectively. Compared to before, the polarization changes from 90° linear polarization to 120° linear polarization in the range of *z* = 7 mm to *z* = 18 mm. It can be observed that the *x* polarization gradually increases while the *y* polarization gradually decreases with the transmission distance. Figure 9c1–c5 and d1–d5 show the experimental results of generated Bessel beams’ *Ex* and *Ey* field distributions in the *x*-*z* plane, which is basically consistent with the simulation results.
